# *Plasmodium falciparum* and *Plasmodium vivax* Prevalence in Ethiopia: A Systematic Review and Meta-Analysis

**DOI:** 10.1155/2019/7065064

**Published:** 2019-12-03

**Authors:** Teshiwal Deress, Mekonnen Girma

**Affiliations:** Unit of Quality Assurance and Laboratory Management, School of Biomedical and Laboratory Sciences, College of Medicine and Health Sciences, University of Gondar, P.O. Box 196, Gondar, Ethiopia

## Abstract

**Background:**

Malaria is a protozoan disease caused by the *Plasmodium* species. Among the five *Plasmodium* species. Among the five *Plasmodium falciparum* and *Plasmodium vivax* malaria are by far the most predominant and widely *distributed in Ethiopia. *Malaria is one of the leading causes of morbidity and mortality globally, particularly in the sub-Saharan countries including Ethiopia. It is also a major obstacle to socio-economic development in the country.

**Methods:**

Articles were searched from PubMed, Google Scholar, and Science Direct databases. The pooled prevalence estimates were analyzed using the DerSimonian-Laird random-effects model and the possible sources of heterogeneity were evaluated through subgroup analysis, metaregression, and sensitivity analysis. Publication bias was analyzed using funnel plots and Egger's test statistics. The data management and analysis were done using STATA 15.1 version software.

**Results:**

Among 922 studies initially identified, thirty-five full-text articles fulfilled the inclusion criteria and included in the study. The combined, *Plasmodium falciparum* and * Plasmodium vivax* malaria are by far the most predominant and widely

**Conclusions:**

This systematic review and meta-analysis showed a high malaria prevalence in Ethiopia. Therefore, previous prevention and control measures should be revised and/or strengthened as appropriate and new strategies should be implemented. In addition, technical, financial and material support, and coordination of the regional capacity building and logistics should be adequately implemented.

## 1. Background

Malaria is a protozoan disease which is transmitted by the female Anopheles mosquito [1]. It is caused by five species of the genus *Plasmodium* [2]. Among these, four species including *P. vivax*,* P. falciparum*,* P. ovale*, and *P. malarae* are known to infect human beings in Ethiopia [3]. However, *P. falciparum* and *P. vivax* are by far the most predominant and widely distributed parasites in Ethiopia [1, 4–7].

Malaria transmission is highly affected by environmental variables such as topography, rainfall, climate, and socio-economic conditions of the population [4, 8–11]. For this reason, tropical regions including Ethiopia with warm temperature, heavy rainfall, and high humidity are conducive for mosquito breeding, longevity and parasite sporogony [12, 13]. In Ethiopia malaria is endemic, unstable [9, 14] and its transmission is seasonal [9–11]. The transmission peaks bi-annually from September to December and April to May, coinciding with the major harvesting seasons [10, 11]. Areas located less than 2,000 meters above the sea level are considered malarious [7]. Regarding clinical manifestations, fever, high temperature, sweating, shivering, vomiting, and severe headache are the peculiar malarial precursors [15]. Currently, several types of malaria diagnostic tests are available including; microscopy, rapid diagnostic tests (RDTs) and polymerase chain reaction (PCR) assays. Although microscopy has limited sensitivity and needs skilled professionals, it remains the gold standard for malaria diagnosis [16]. The Ethiopian government has adopted various strategies to control malaria including early diagnosis, prompt treatment, selective vector control, and epidemic prevention [8].

Malaria causes severe complications, severe anemia, acute renal failure, and hypoglycemia [15]. It is one of the leading causes of morbidity and mortality with an enormous medical and economic impact [1, 3, 4, 17]. An estimated 3.3 billion people are at risk of malaria worldwide [17]. Particularly it is a major problem in the tropical and subtropical regions [18]. Though malaria is declining globally, still it is a major challenge for the public health and socio-economic development particularly in sub-Saharan Africa including Ethiopia [19]. During 2016, about 445,000 deaths occurred due to malaria infection of which about 91% were from the African region [20]. Later in 2017, there were an estimated 219 million malaria cases and 435,000 deaths globally. From this, 92% of the cases occurred in sub-Saharan Africa [21]. Malaria control and elimination are extremely challenging and resource-intensive. For this reason, an estimated 3.1 billion dollar was invested globally during 2017 [21]. Despite considerable progress in malaria control measures, it remains the major public health problem in Ethiopia where an estimated 68% of the population lives in malarious regions [7, 22–25]. The unstable malaria transmission patterns make Ethiopia prone to multifocal epidemics which can cause catastrophic public health emergencies [7]. Over five million malaria cases and thousands of deaths happen annually in Ethiopia. It is also a major obstacle for the socio-economic development of the country as the major malaria transmission period coincides with the peak agricultural activities [25]. Therefore, the aim of this study was to determine the pooled prevalence of *P. falciparum* and *P. vivax* infections in Ethiopia among studies conducted from 2009 to 2018.

## 2. Methods

### 2.1. Study Area

The study was conducted in Ethiopia which has a total area of 1.1 million square kilometers [7, 26]. The country's topographic feature ranges from 110 meters below sea level to 4,550 meters above sea level. The predominant climate type is tropical monsoon, with three broad (lowland, midland, and highland) agro-ecological regions [26]. The mean annual temperatures range from 10°C to 16°C in the “highlands,” 16°C to 29°C in the “midlands,” and 23°C to 33°C in the “lowlands.” The highlands and lowlands receive annual rainfalls ranging from 500 metersm to over 2,000 metersm and from 300 metersm to 700 metersm, respectively [7]. Currently, the Ethiopian population is estimated to be more than 90 million from which about 68% is living in malaria risk areas [27].

### 2.2. Literature Search Strategy

This systemic review and meta-analysis was conducted using published studies on the prevalence of *P. falciparum* and *P. vivax* malaria parasites in Ethiopia. Our literature search strategy, selection of publications, and reporting of results were conducted according to the PRISMA (Preferred Reporting Items for Systematic Reviews and Meta-analysis) guidelines [28]. Articles searched using a combination of search terms and Boolean functions. TD and Kasaw Adane searched the PubMed, Google Scholar, and Science Direct databases. Though the search strategy differs from database to database, we commonly used [(“*Plasmodium falciparum*” OR “*Plasmodium vivax*” OR “malaria”) AND (“prevalence” OR “epidemiology”) AND “Ethiopia”] rule of combination to obtain relevant articles. In addition, manual Google searching and screening of reference lists of the included studies were done to access additional articles. Articles were searched without any time restriction until April 4, 2019, however, all the eligible studies were published from 2009 to 2018.

### 2.3. Eligibility Criteria

Article searching was not restricted by publication year; however, only English version full-text articles were considered. We include only primary studies published in peer-review journals, thereby excluding reviews, letters, short communications, posters, studies conducted through clinical examination only, and conference abstracts. All types of study designs among patients of any age groups reported malaria prevalence using microscopy or rapid diagnostic tests (RDT) in the Ethiopian settings were included. In addition, key qualitative findings were included in the systematic review.

### 2.4. Article Selection and Data Extraction

All searched articles were imported into the EndNote X9 version software and then duplicate files were removed. Both authors independently screened articles by title, abstract and full text to identify potentially eligible studies according to the predetermined inclusion criteria. After that, authors of this article developed the data extraction form in Microsoft Excel Spreadsheet and then data were extracted from full-text articles. The data extraction sheet included the name of the first author, year of publication, region (province), geographic location (Highland, midland or lowland), study group (subjects), study design, sample size, sampling technique, diagnostic method, total positive finding, and species-specific total positive finding. In addition, major findings were extracted qualitatively for the systematic review. Then, the extracted data files from the two investigators were systematically checked for consistency, and any inconsistencies were resolved by discussion. In addition, Mr. Kasaw Adane revised the data abstraction.

### 2.5. Data Synthesis and Analysis

The studies' proportion (*p*) and their standard error (*se*) were calculated using *p* = *r*/*n* and $se=\sqrt{p\left(1-p\right)/n}$, respectively, where *r* stands for the number of positive individuals for malaria and *n* represents the sample size for malaria prevalence study. However, to normalize the distribution, study level estimates were logit transformed logit *p* = ln[*p*/(1 − *p*)], where ln is the natural logarithm. The standard error (*se*) of logit event estimate was calculated as $se=\sqrt{1/r+1/\left(n-r\right)}$. In situations with high across study heterogeneity, the use of random-effects models is recommended as it produces study weights which primarily reflects between-study variation [29]. The *I*^2^ statistics estimates the presence of observed difference between-studies due to heterogeneity rather than by chance and it can range from 0 to 100%. The 25%, 50%, and 75% values represent low, medium, and high heterogeneity between studies, respectively [30]. This meta-analysis was fitted with the random-effects model as *I*^2^ was 100% which is a definite indicator of considerable heterogeneity between studies. The overall and sub-group prevalence estimates were computed using the Der Simonian-Laird (DL) model [31]. A *p*-value of less than 0.05 was used to declare the presence of heterogeneity. In addition, subgroup analysis, meta-regression, and publication bias were conducted. Data manipulation and statistical analysis were done using STATA 15.1 version software.

### 2.6. Quality Assessment

The quality of the included studies was assessed using the Joanna Briggs Institute (JBI) quality assessment tool for the prevalence studies [32]. The evaluation criteria included nine parameters; (1) appropriate sampling frame, (2) proper sampling technique, (3) adequate sample size, (4) study subject and setting description, (5) sufficient data analysis, (6) use of valid methods for the identified conditions, (7) valid measurement for all participants, (8) using appropriate statistical analysis, and (9) adequate response rate. Both authors assessed the quality of included studies. Finally, studies with a total score of ≥50% were considered as having a low risk of bias.

## 3. Results

### 3.1. Study Selection

**Figure 1:**
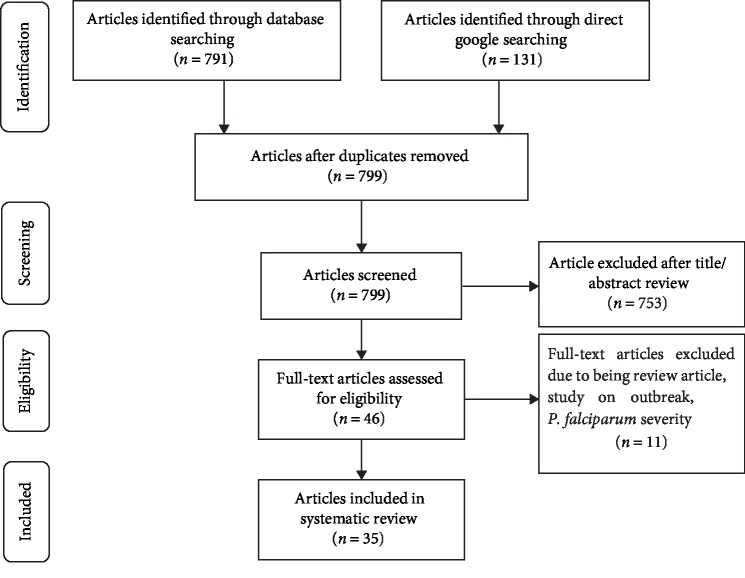
The PRISMA flow diagram showing the study selection process, 2019.

Initially, 922 studies were retrieved from the database and manual searching. Among these, 123 studies were excluded due to duplication. From the remaining 799 articles, 753 of them were excluded after evaluation of their title and abstract confirming nonrelevance to this study. Further 46 articles were screened and 11 full-text articles were excluded due to being review article, studies on outbreaks, and *P. falciparum* severity (Figure 1). Finally, a total of 35 full-text articles were included in the study.

### 3.2. Characteristics of the Included Studies

A total of thirty-five articles [1, 4, 23, 25, 33–63] were included in this systematic review and meta-analysis. Overall 1,055,155 study subjects were diagnosed for malaria infection from the thirty-five included studies. Among this, 263,910 were positive which accounted for 149,142, 107,236, and 8,099 for *P. falciparum*, *P. vivax*, and mixed infections, respectively. Regarding study design, all the included studies were cross-sectional studies and their sample size ranged from 204 from Southern Nations and Nationalities Peoples Region (SNNPR) [62] to 807, 275 data collected from multiple (mixed) regions [35]. Nearly all studies were conducted by microscopy which is currently a gold standard for malaria diagnosis. Most studies were obtained from the Amhara (34.3%) and Oromia (28.6%) regions; however, there was no study found from Tigray, Afar, Somali, Gambella, and Harari regions (Table 1). Regarding qualities of the included studies, all studies were evaluated with nine criteria of the JBI quality assessment tool for the prevalence studies and all of them were having a low risk of bias.

### 3.3. Qualitative Findings

Although malaria is decreasing, it is still a major public health problem in Ethiopia [1, 23, 60]. Malaria prevalence was extremely high [36] and the predominant species are *P. vivax* and *P. falciparum* [60]. Studies indicated that *P. falciparum* prevalence was higher than *P. vivax* [1, 23, 38, 48, 64–66]. However, in some areas, *P. vivax* was the predominant species [4, 54, 60, 67]. Low prevalence of malaria was observed in the highland-fringes [67]. With respect to vulnerable age groups, the productive age group (15–45 years) were highly vulnerable to malaria [1, 4, 45]. However, other studies indicated that children in the age group of <15 years were the most affected group [34, 47, 65, 66]. The highest malaria transmission was reported during the spring season [1, 45, 47]. Regarding gender preference, men were more affected than female [23]. Concerning contributory factors, large-scale irrigation [68], type of house construction, insecticide chemicals spray and residing to nearby stagnant water [38], proximity to mosquito breeding sites [65], educational level, outdoor sleeping, and lack of bed net utilization [36] were identified as a factor for malaria infection.

### 3.4. Combined Prevalence of Malaria in Ethiopia

**Figure 2:**
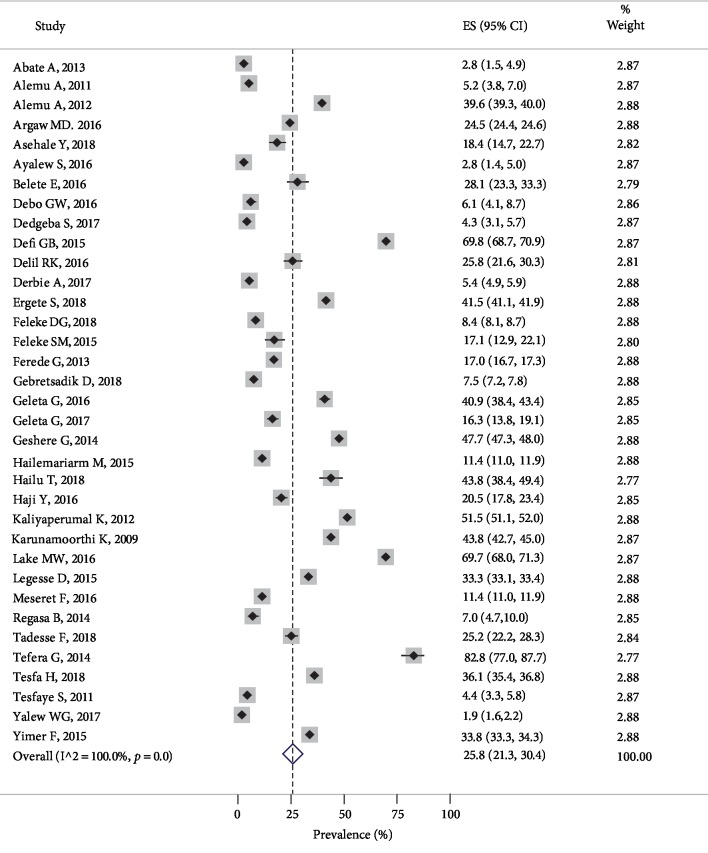
Forest plot of the combined malaria pooled prevalence estimate in Ethiopia, 2019.

Malaria prevalence in Ethiopia ranged from 1.9% from the Amhara region [63] to 82.8% from the SNNPR [62]. The rend analysis indicated that from 2009 to 2014 malaria prevalence was kind of trend off appearance characterized by high prevalence followed by low prevalence estimates ([Supplementary-material supplementary-material-1]). From 2015 to 2017 almost it was decreasing however again during 2018 it indicated an increment of the prevalence. Since, there was high heterogeneity among the studies (*I*^2^ = 100%, *p* = 0.0). The DerSimonian-Laird random-effects model at 95% was fitted. The pooled prevalence of malaria was found to be 25.8% (95% CI [21.3, 30.4]. The forest plot for the pooled estimate and prevalence of each included study with the corresponding CI is indicated in Figure 2.

### 3.5. Plasmodium falciparum Prevalence

**Figure 3:**
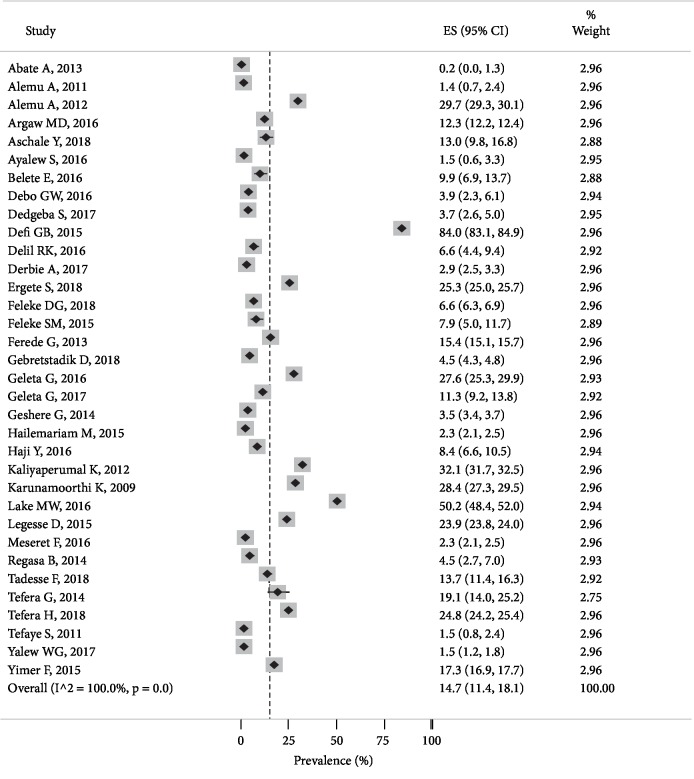
The figure shows the pooled prevalence of *P. falciparum* malaria in Ethiopia, 2019.

The prevalence of *P. falciparum* among the primary studies ranged from 0.2% in the Amhara region [33]to 84% in the Oromia region [41]. *Plasmodium falciparum* was the dominant parasite that accounted for 14.7% of pooled prevalence estimate with 95% CI (11.4, 17.1) and *I*^2^ = 100% between-study heterogeneity (Figure 3).

### 3.6. Plasmodium vivax and Mixed Infections Prevalence

**Figure 4:**
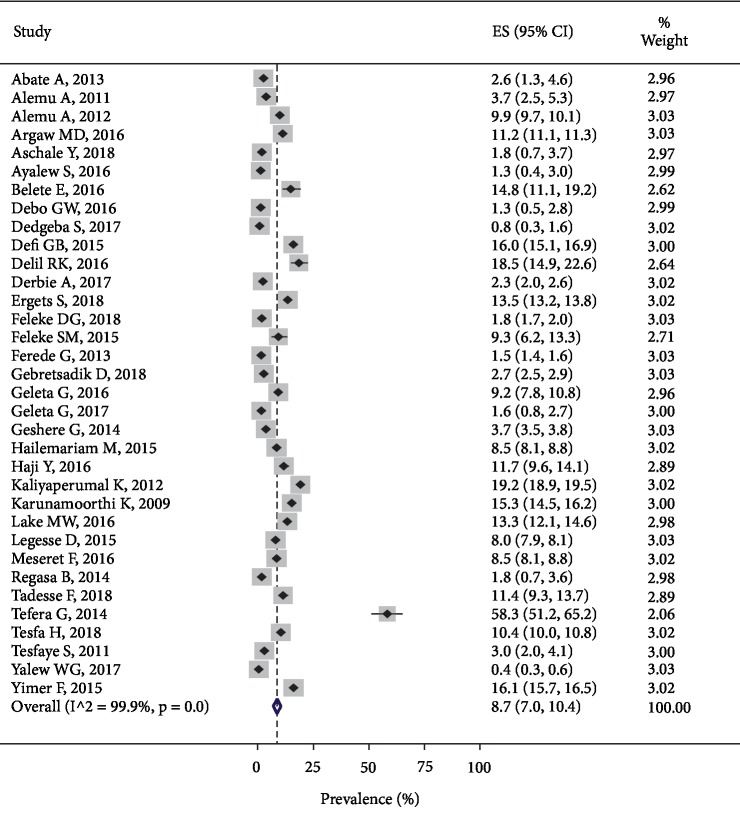
The figure shows the pooled prevalence of *P. vivax* malaria in Ethiopia, 2019.

In this systematic review and meta-analysis, the least (0.4%) [63] and the highest (58.3%) [62] *P. vivax* prevalence estimates were obtained from the Amhara and SNNP regions, respectively. Currently, *P. vivax* is the second most dominant malaria parasite in Ethiopia which accounted for 8.7% with 95% CI (7.0, 10.4). Similarly, the pooled estimate of the *P. falciparum* and *P. vivax* coinfections was 1.2% with 95% CI (0.38, 0.88) and *I*^2^ = 99.63% level of heterogeneity (Figure 4).

### 3.7. Investigation of Heterogeneity

Heterogeneity in systematic review and meta-analysis is inevitable due to differences in methodology, sample size and sampling technique, data collection period, and study participant characteristics. In this meta-analysis, the possible sources of the heterogeneity were assessed using subgroup analysis, sensitivity analysis, and meta-regression. From the regional subgroup analysis of the overall malaria prevalence, the highest overall pooled estimate was found from Oromia region (44.5%) followed by SNNP region (28.5%) and the list prevalence (19.2%) was documented in the Amhara region. On the other hand, from the agro-ecological regions subgroup analysis, the highest subgroup prevalence estimate was obtained from the mixed region studies (37.6%) followed by midland (26%). The list subgroup prevalence was unexpectedly among studies from the lowland regions (20.7%) (Table 2).

In addition, a sensitivity test was done to identify the influence of each study and the result indicated no influence on the pooled prevalence estimate of malaria, while removing one study at a time from the analysis. The result of the meta-regression analysis indicated no significant relationship between the pooled prevalence of malaria with a year of a publication and sample sizes of studies (Table 3).

### 3.8. Assessment of Publication Bias

The presence of publication bias was evaluated subjectively using funnel plots and objectively using the Egger's test. Each point in funnel plots represented a separate study and asymmetrical distribution is evidence of publication bias [69]. First, studies' effect sizes were plotted against their standard errors and the visual evaluation of the funnel plots indicated that in all cases the funnel plots were slightly asymmetrical ([Supplementary-material supplementary-material-1]); however, the subjective evidence from the funnel plots was objectively evaluated using Egger's weighted regression statistics. According to the symmetry assumptions, there was no publication bias in the combined (*p* = 0.84), *P. falciparum* (*p* = 0.73), *P. vivax* (*p* = 0.15), and mixed (*p* = 0.68) infections pooled prevalence estimates.

## 4. Discussion

This systematic review and meta-analysis was conducted using thirty-five full-text articles to determine the pooled prevalence of *P. falciparum *and* P. vivax* infections in Ethiopia. Despite the declining of malaria infections in Ethiopia, the disease still remains one of the leading causes of morbidity and mortality affecting all age groups [1, 3, 4, 17]. It can cause severe anemia, hypoglycemia, renal failure [15], loss of productivity, school absenteeism, and other complications [1]. So, accurate malaria prevalence data are vital for the proper diagnosis, treatment, prevention/control, and policy formulation [70].

The combined malaria prevalence trend analysis among primary studies did not uniformly change overtime. From 2009 to 2014 the prevalence was characterized by an abrupt change in either direction (high or low). From 2015 to 2017 the prevalence showed a slight decrement compared to the previous years ([Supplementary-material supplementary-material-1]). However, during 2018 the prevalence showed an increment. The inconsistency in prevalence estimates over time could be due to the fact that malaria infection in Ethiopia is highly variable and unstable and the occurrence of epidemics over several locations (agro-ecological regions) of the country.

In this meta-analysis, the combined estimated pooled prevalence of *P. falciparum* and *P. vivax* infections was 25.8%, which is lower than 32%, the estimated pooled prevalence from the sub-Saharan Africa countries [71]. Similarly, the pooled prevalence estimates of *P. falciparum* (14.7%) and *P. vivax* (8.7%) parasites resulted in a proportion of 62.8% and 37.2%,respectively. These results were nearly similar to the previous predictions of *P. falciparum* (60%) and *P. vivax* (40%) in Ethiopia [25, 62, 72, 73]. However, the estimates contradict to 64% and 34% of *P. vivax* and *P. falciparum*, respectively from India [74].

Malaria prevalence in Ethiopia is seasonal [34, 36, 47, 59] with its transmission peaks bi-annually from April to May and from September to December that coincides with the peak agricultural activities [34, 47, 73, 75]. Due to this reason, the country's economy is significantly affected. Not only this, but malaria transmission is also highly variable [34, 36, 47, 59]. This could be due to the presence of different topographic platforms that control the multiplication rate and diversity of the Anopheles mosquito vectors.

Several years ago, the distribution of malaria was largely determined by altitude which means malaria was restricted in the low lands of Ethiopia because the temperature is very important for the parasite multiplication. However, currently there is a paradigm shift that malaria becomes predominate in the midlands and even it is commonly found in the highlands of Ethiopia where it did not exist previously [76–82]. This could be explained by the fact that currently there is increased industrialization and deforestation that makes a change in the highlands temperature over time which in turn create optimum temperature for the parasite multiplication. The results of this meta-analysis also confirmed that the highest prevalence estimate of malaria was obtained from studies conducted on mixed agro-ecological regions of low lands and midlands (37.6%) followed by 26% from the midlands and the least (20.7%) was unexpectedly from the low land regions. This could be due to changes in the epidemiological transmissions of malaria from the low lands to the high lands because of increased temperature in the highlands of Ethiopia.

Regarding the degree of susceptibility of the age groups, some studies established that age-groups less than 15 were highly affected by malaria parasites [34, 47, 65, 66]. However, other reports confirmed that the productive age groups (15–45 years) were highly affected than other age groups [1, 4, 45]. This result was supported with the pan African health organization report which established that malaria prevalence was highest among 20–39 age groups [70]. This discordant report on the degree of malaria susceptibility of age groups could be attributed due to study subject susceptibility differences because of genetic variation or other lifestyle characteristics. However, it requires additional facts or strong justifications to determine the specific age group that is more susceptible to malaria infection. Anyways, the high malaria prevalence in Ethiopia, seasonal transmission pattern particularly during the peak agricultural activities, and the more productive age group susceptibility can significantly affect the socio-economic growth of the country. In addition, urbanization is expected to reduce malaria transmission; however, in this study, there was no significant difference in the prevalence estimates between the urban area and rural areas. The possible explanation could be malaria vector adaptation to urban areas, ditches, and urban agriculture practices could contribute to the high prevalence of malaria in the urban areas.

In this meta-analysis, there was high heterogeneity among the included studies. We have tried to assess the possible sources of heterogeneity through subgroup analysis, meta-regression, and sensitivity analysis; however, in all cases, the sources of variability were not identified. The most likely reason for this huge heterogeneity is that some of the studies were obtained from the highly malarious areas of the country while others were obtained from medium and low malaria-risk regions. In addition, studies were conducted in different malaria transmission seasons which means that some studies were conducted during the high transmission periods while others were conducted during the least transmission seasons or the combination that could be a significant contributing factor for the high heterogeneity.

## 5. Limitations

The inclusion of articles published only in the English language can affect the pooled prevalence estimate. As the included studies were cross-sectional designs the outcome variable could be affected by other confounding variables. In addition, the included studies were not proportionally distributed throughout the country. More than one-third of the studies were obtained from the Amhara region. However, no study obtained from Benishangul Gumuz, Tigray, Gambella, and Afar regions. Besides subgroup analysis was conducted only for the combined malaria prevalence estimate.

## 6. Conclusions and Recommendations

This systematic review and meta-analysis showed a high prevalence of malaria in Ethiopia. Therefore, the previous prevention and control measures should be revised and/or strengthened as appropriate, and new strategies should be implemented. In addition, technical, financial, and material support to regions, and coordination of the regional capacity building and logistics by the Ministry of Health should be adequately implemented. Further, it will be more effective if separate and stand-alone malaria prevention and control task forces organized in all regions of the country.

## Figures and Tables

**Table 1:** Included studies in the meta-analysis of malaria parasite prevalence in Ethiopia, 2019.

First author, reference	Year	Region	Altitude	Study group	Sample	Positive	P.F.	P.V.	Mixed	Method of diagnosis
Abate [33]	2013	Amhara	Lowland	Suspected	425	12	1	11	0	Microscopy
Alemu [34]	2011	Oromia	Midland	Suspected	804	42	11	30	1	Microscopy
Alemu [1]	2012	Amhara	Midland	Suspected	59208	23473	17605	5868	75	Microscopy
Argaw [35]	2016	Mixed	Mixed	Suspected	807275	198066	99826	90318	8121	Microscopy
Aschale [36]	2018	Amhara	Lowland	Migrants	385	71	50	7	14	Microscopy
Ayalew [37]	2016	Amhara	Midland	Suspected	392	11	6	5	0	RDT
Belete [38]	2016	SNNPR	Midland	Suspected	324	91	32	48	11	Microscopy
Debo [39]	2016	SNNPR	Midland	Pastoralist	461	28	18	6	4	Microscopy
Dedgeba [40]	2017	SNNPR	Mixed	Suspected	1007	43	8	35	0	Microscopy
Defi [41]	2015	Oromia	Midland	Suspected	6831	4768	4,004	764	0	Mixed
Delil [42]	2016	SNNPR	Midland	Suspected	411	106	27	76	3	Microscopy
Derbie [43]	2017	Amhara	Midland	Suspected	8057	434	233	184	17	Microscopy
Ergete [44]	2018	SNNPR	Mixed	Suspected	54160	22,494	13728	7297	1469	Microscopy
Feleke [45]	2018	Amhara	Midland	Suspected	31810	2670	2087	557	26	Microscopy
Feleke [46]	2015	Oromia	Midland	Suspected	280	48	22	26	0	Microscopy
Ferede [47]	2013	Amhara	Lowland	Suspected	55833	9486	8602	852	32	Microscopy
Gebretsadik [48]	2018	Amhara	Midland	Suspected	27492	2066	1243	734	89	Microscopy
Geleta [49]	2016	Benishangul	Lowland	Children	1523	623	420	63	140	Microscopy
Geleta [50]	2017	Benishangul	Lowland	Pregnant	760	124	86	12	23	Microscopy
Geshere [51]	2014	Oromia	Mixed	Suspected	77534	36966	1303	1357	0	Microscopy
Hailemariam [52]	2015	Oromia	Highland	Suspected	22025	2521	499	1865	157	Microscopy
Hailu [53]	2018	Amhara	Lowland	Children	333	146	0	0	0	Microscopy
Haji [54]	2016	Oromia	Mixed	Suspected	830	170	70	97	3	Microscopy
Karunamoorthi [25]	2009	Oromia	Mixed	Suspected	6863	3009	1946	1052	11	Microscopy
Karunamoorthi [55]	2012	Oromia	Mixed	Suspected	51610	26602	16584	9913	105	Microscopy
Lake [56]	2016	Amhara	Midland	Suspected	2958	2062	1484	392	185	Mixed
Legesse [57]	2015	SNNPR	Mixed	Suspected	317867	105755	75929	25329	4497	Microscopy
Meseret [58]	2016	Oromia	Highland	Suspected	22025	2521	499	1866	157	Microscopy
Regasa [59]	2014	Mixed	Mixed	Suspected	400	28	18	7	3	Microscopy
Tadesse [60]	2018	Oromia	Midland	Suspected	810	204	92	111	1	Microscopy
Tesfa [61]	2018	Amhara	Highland	Suspected	20483	7428	5115	2139	174	Microscopy
Tefera [62]	2014	SNNPR	Midland	Suspected	204	169	39	119	11	Microscopy
Tesfaye [4]	2011	SNNPR	Midland	Suspected	1082	48	16	32	0	Microscopy
Yalew [63]	2017	Amhara	Mixed	Suspected	7878	159	102	35	4	RDT
Yimer [23]	2015	SNNPR	Midland	Suspected	34060	11523	5888	5485	150	Microscopy

Key: PF, *P. falciparum*, PV, *P. vivax*, mixed may be mixed regions, RDT: rapid diagnostic test, mixed altitude or mixed diagnosis using microscopy, RDT.

**Table 2:** Subgroup analysis of the possible heterogeneity sources for the overall pooled prevalence of malaria in Ethiopia, 2019.

Heterogeneity source	Category	Prevalence (95% CI)	*I* ^2^ (%)	*P*-value
Region	Oromia	44.5 (21, 51)	100	0.0
	Amhara	19.2 (12, 26)	100	0.0
	SNNPR	28.5 (22, 35)	99.9	0.0
	Others	22 (12, 33)	99.2	0.0

Altitude	Lowland	20.7 (15, 26)	99.7	0.0
	Midland	26 (16, 34)	100	0.0
	Mixed	37.6 (30, 45)	100	0.0
	Unidentified	24 (10, 39)	100	0.0

Publication year	2009–2012	40.6 (28, 53)	99.9	0.0
	2013–2015	27.9 (19, 37)	100	0.0
	2016–2018	19.3 (14,25)	100	0.0

Sampling technique	Probability	15.5 (3, 28)	99.9	0.0
	Survey	26.6 (19, 35)	100	0.0
	Unknown	34.4 (16, 53)	99.9	0.0
	Nonprobability	35.9 (35, 36)	—	—

Setting	Rural	32.3 (27, 38)	99.9	0.0
	Urban	27.6 (4, 51)	—	—
	Mixed	28.3 (21, 36)	100	0.0
	Unknown	15.9 (7, 25)	99.9	0.0

Study participant	General population	25.1 (20, 30)	100	0.0
	Specific groups	29.5 (11, 46)	99.8	0.0

Diagnostic method	Microscopy	24.5 (20, 29)	100	0.0
	RDT	1.9 (2, 2)	99.8	0.0
	Mixed	69.8 (69, 71)	—	—

**Table 3:** A meta-regression analysis of factors for heterogeneity of the prevalence of malaria parasites in Ethiopia, 2019.

Prevalence estimate	Heterogeneity source	Coefficients	Std. error	*P*-value
Combined	Publication year	−0.121798	0.0954201	0.211
	Sample size	1.06	1.62	0.516

PF	Publication year	−0.110215	0.105692	0.305
	Sample size	1.23	1.75	0.487

PV	Publication year	−0.1352612	0.0874682	0.132
	Sample size	1.17	1.45	0.425

Mixed	Publication year	0.0947128	0.1555648	0.549
	Sample size	1.72	1.74	0.540

## Data Availability

The data used to support the findings of this study are included within the articled and attached as supplementary material.

## Abbreviations

*P. vivax*: *Plasmodium vivax*
*P. falciparum*: *Plasmodium falciparum*
RDT: Rapid diagnostic tests
PRISMA: Preferred reporting items for systematic reviews and meta-analysis
JBI: Joanna briggs institute
SNNP: Southern nations and nationalities peoples
CI: Confidence interval.

## Supplementary Materials

Supplementary 1 Figure S1: Time trend analysis of malaria prevalence in Ethiopia from 2009 to 2018. Figure S2: funnel plots of logit event estimated (logitp) of combined (a), P. falciparum (b), P. vivax (c), and mixed infections (d) in Ethiopia, 2019.
