# Supplementary material for: Plasmodium falciparum and Plasmodium vivax Prevalence in Ethiopia: A Systematic Review and Meta-Analysis
Source: Malar Res Treat. 2019 Dec 3;2019:7065064. doi: 10.1155/2019/7065064 (PMC7024085; doi:10.1155/2019/7065064)
Supplement: Supplementary 1 — Figure S1: Time trend analysis of malaria prevalence in Ethiopia from 2009 to 2018. Figure S2: funnel plots of logit event estimated (logitp) of combined (a), P. falciparum (b), P. vivax (c), and mixed infections (d) in Ethiopia, 2019. [file 7065064.f1.pdf]

Fig. S1. Malaria prevalence trend analysis in Ethiopia

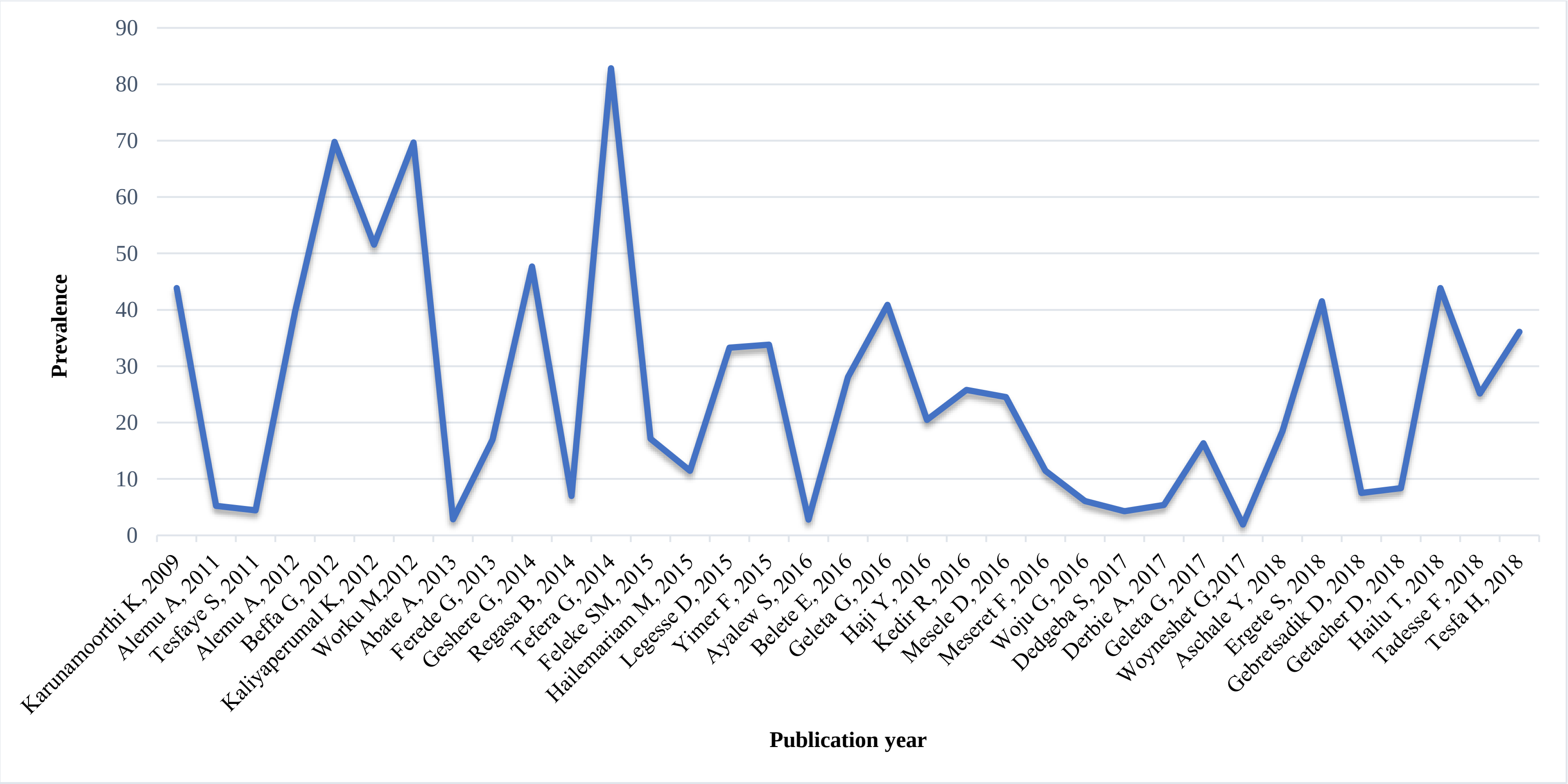

FIGURE: Time trend analysis of malaria prevalence in Ethiopia from 2009 to 2018.

Fig. S2. Funnel plots of malaria prevalences in Ethiopia

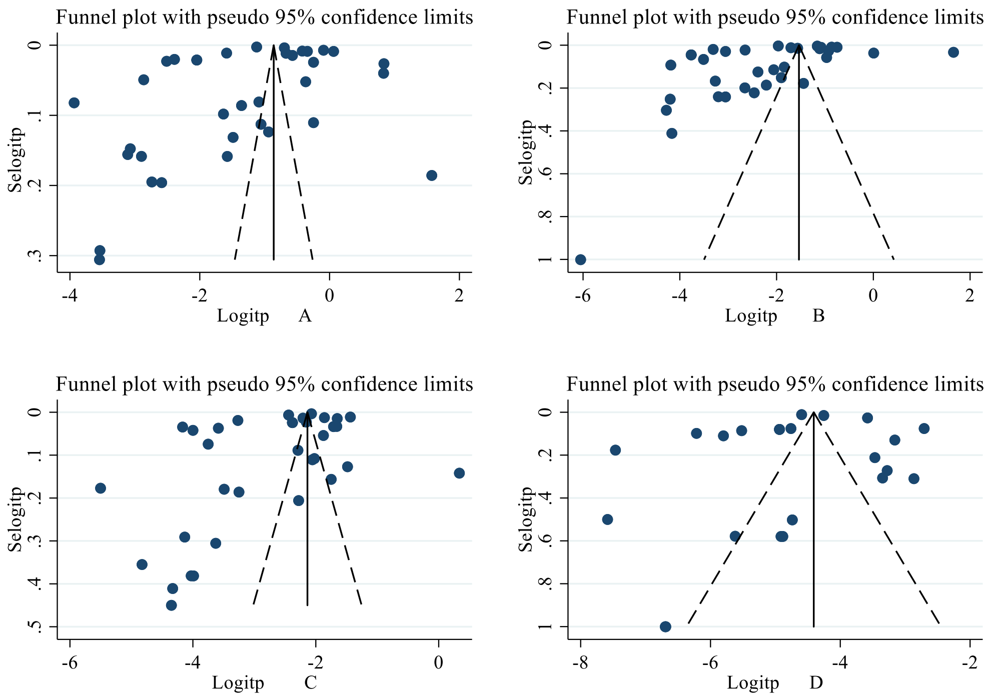

FIGURE**:** Funnel plots of logit event estimated (logitp) of combined (A), *P. falciparum* (B), *P. vivax* (C), and mixed infections (D) in Ethiopia, 2019.
